# Estimating the Comparative Effectiveness of Feeding Interventions in the Pediatric Intensive Care Unit: A Demonstration of Longitudinal Targeted Maximum Likelihood Estimation

**DOI:** 10.1093/aje/kwx213

**Published:** 2017-06-24

**Authors:** Noémi Kreif, Linh Tran, Richard Grieve, Bianca De Stavola, Robert C Tasker, Maya Petersen

**Affiliations:** 1 Centre for Health Economics, University of York, York, United Kingdom; 2 Division of Biostatistics, School of Public Health, University of California, Berkeley, Berkeley, California; 3 Centre for Statistical Methodology, London School of Hygiene and Tropical Medicine, London, United Kingdom; 4 Department of Health Services Research and Policy, London School of Hygiene and Tropical Medicine, London, United Kingdom; 5 Department of Medical Statistics, London School of Hygiene and Tropical Medicine, London, United Kingdom; 6 Department of Anesthesiology, Perioperative and Pain Medicine, Division of Critical Care, Boston Children’s Hospital, Boston, Massachusetts; 7 Department of Neurology, Boston Children’s Hospital, Boston, Massachusetts

**Keywords:** causal inference, epidemiologic methods, longitudinal targeted maximum likelihood estimation, machine learning, Super Learner, time-dependent confounding

## Abstract

Longitudinal data sources offer new opportunities for the evaluation of sequential interventions. To adjust for time-dependent confounding in these settings, longitudinal targeted maximum likelihood based estimation (TMLE), a doubly robust method that can be coupled with machine learning, has been proposed. This paper provides a tutorial in applying longitudinal TMLE, in contrast to inverse probability of treatment weighting and g-computation based on iterative conditional expectations. We apply these methods to estimate the causal effect of nutritional interventions on clinical outcomes among critically ill children in a United Kingdom study (Control of Hyperglycemia in Paediatric Intensive Care, 2008–2011). We estimate the probability of a child’s being discharged alive from the pediatric intensive care unit by a given day, under a range of static and dynamic feeding regimes. We find that before adjustment, patients who follow the static regime “never feed” are discharged by the end of the fifth day with a probability of 0.88 (95% confidence interval: 0.87, 0.90), while for the patients who follow the regime “feed from day 3,” the probability of discharge is 0.64 (95% confidence interval: 0.62, 0.66). After adjustment for time-dependent confounding, most of this difference disappears, and the statistical methods produce similar results. TMLE offers a flexible estimation approach; hence, we provide practical guidance on implementation to encourage its wider use.

Large observational databases such as electronic health records are increasingly being used to answer questions of comparative effectiveness. The longitudinal structure of these data sets allows researchers to estimate the effects of interventions that change over time. Examples include the treatment of chronic diseases such as diabetes and hypertension, where decisions such as when to initiate a treatment, change the dose, or introduce a concomitant medication are repeatedly updated over time. For decision-makers to compare the consequences of alternative longitudinal interventions, it is essential to carefully define the strategies of interest (1). A static regime for time-varying interventions prespecifies the full sequence of interventions, irrespective of changing patient characteristics over time (e.g., “always treat”). Dynamic regimes, or individualized treatment rules, in contrast, define a set of rules as a function of time-varying patient characteristics (2–6). While sequentially randomized trials provide an ideal design for the evaluation of dynamic regimes (7, 8), such trials are still relatively rare (9) and are impractical for many clinical and health policy questions (10).

Where the only available data for estimating the treatment effects of interest are from observational studies, statistical methods are required to address both baseline and time-dependent confounding. The latter arises in longitudinal settings, when the uptake of treatment may depend on factors that influence the outcome and are also affected by earlier treatments. It is widely recognized that standard regression analysis cannot deal with time-dependent confounding (2, 11). While in the last few decades progress has been made in developing appropriate statistical methods for addressing time-varying confounding (see Daniel et al. (12) for a review), applications of these approaches have been confined to relatively few clinical areas, such as human immunodeficiency virus infection (13). Further methodological and applied research that demonstrates these approaches for handling time-varying confounding in different contexts is required.

Inverse probability of treatment weighting (IPTW) (11, 14), a simple and intuitive method for estimating the effect of time-varying treatments, results in unstable and biased estimates in the presence of data sparsity, even when weights are normalized and extreme weights are truncated (15). A commonly used alternative approach, parametric g-computation (2), requires parametric specification of multiple aspects of the data distribution, including models for the full conditional densities (or probability distributions) of the outcome and of the time-dependent confounders given the past. Similarly, structural nested mean models require a parametric model for the treatment effect, the “blip function” (16). A perennial concern with these approaches is that they are prone to model misspecification, leading to biased estimates of treatment effects.

Targeted learning (17) has been proposed as a general approach to estimation of a range of causal parameters for causal inference problems with both time-constant and time-varying interventions (18–22). Targeted learning encompasses a semiparametric, doubly robust estimation approach, targeted maximum likelihood estimation (TMLE) (17) for single-time-point and longitudinal causal effects. TMLE combines estimates of the treatment and outcome mechanisms and provides a consistent estimator of the target parameter if either the treatment or the outcome mechanism is estimated consistently. If both are estimated consistently, TMLE is efficient (19). In order to reduce bias, achieve efficiency, and ensure accurate statistical inference, TMLE is often coupled with machine learning—particularly the Super Learner, a cross-validation-based estimator selection approach (23, 24). (For a tutorial on single-time-point interventions, see, for example, Schuler and Rose (25).)

In this paper, we demonstrate the application of the longitudinal TMLE estimator based on iterative conditional expectations (21, 26) and highlight how it is related to the IPTW (11) and g-computation (2, 26) estimators. While prior studies demonstrating the application of this estimator in longitudinal settings exist (22, 26–30), few observational data applications have discussed the use of the estimator to study the effects of dynamic regimes or subject-responsive adaptive treatment strategies (31).

We apply IPTW, g-computation based on iterative conditional expectations, and longitudinal TMLE to an empirical study investigating an unanswered question of high relevance to clinical decision-makers: What is the optimal timing and quantity of caloric intake for critically ill children? We reanalyze data from a clinical trial, the Control of Hyperglycemia in Paediatric Intensive Care (CHiP) Study (32), to estimate the effect of alternative treatment regimes on the probability of being discharged from the pediatric intensive care unit (PICU) by a given day, under a range of clinically relevant treatment regimes. We follow the general targeted estimation road map (33) to formulate the hypothetical regimes, define the causal parameters of interest, and discuss how to identify, estimate, and interpret these parameters.

## METHODS

### Research question in the CHiP Study

An important objective of critical-care medicine is to provide the appropriate level of nutritional support over the course of the patient’s hospital stay. In most critical-care settings, the preferred mode of nutritional support is the nasogastric (enteral) tube, which is often complemented with intravenous (parenteral) feeding. For adult patients admitted to critical-care units, evidence-based guidelines exist, and in a recent randomized controlled trial, McClave et al. (34) reported that starting nutritional support early after admission to the PICU favorably altered outcomes. For critically ill children, guidelines for nutritional support are limited by the lack of available evidence (35). While a recent randomized trial of nutritional support for children admitted to the PICU found that delaying parenteral nutrition led to favorable clinical outcomes (36), there is no randomized trial evidence to address more complex but important questions, such as the optimal timing and total quantity of nutritional support for children admitted to the PICU.

In recently published findings from the CHiP Study, a randomized trial with 1,369 participants aged ≤16 years (recruited between 2008 and 2011) undertaken at 13 research centers in the United Kingdom, Macrae et al. (32) found that tight glycemic control in critically ill children had no effect on the primary clinical outcome, the number of ventilator-free days. We undertook a secondary analysis of the CHiP data set to investigate the causal effect of different levels of nutritional support on a clinical outcome. We focused on the subgroup of children who were admitted to the PICU to undergo cardiac surgery and were younger than 3 years of age.

We followed the US National Academy of Medicine (formerly Institute of Medicine) guidelines (37) in standardizing individual caloric intake by dividing the individual measures of daily intake by a target level specific to the patient’s sex, age, height, and weight. We defined a patient as “fed” on a given day if he or she received at least 20% of the individualized target. The outcome of interest was being discharged alive from the PICU to other hospital wards by a given day.

The effect of a feeding strategy on a given day on the patient’s discharge status is subject to potential confounding from baseline characteristics, such as age, sex, weight, height, Risk Adjustment for Congenital Heart Surgery (RACHS-1) risk score (which expresses the severity of the child’s condition at admission (38)), and randomization arm (tight or standard glycemic control). Younger children and those with higher risk scores tend to be fed less aggressively and are also likely to stay longer in the PICU, potentially biasing the effect of feeding (as compared with no feeding) towards a seemingly protective effect. While all patients are mechanically ventilated at baseline, being taken off mechanical ventilation is a strong predictor of discharge in the next few days. Patients just taken off mechanical ventilation can, for safety reasons, be fed only by the parenteral (not the enteral) route, making it less likely that their caloric intake will reach the 20% threshold. Hence, a lack of adjustment for mechanical ventilation status could make no feeding appear beneficial. Further time-varying potential confounders include renal replacement therapy, infection, and a vasoactive inotrope score (39).

### Observed data structure

For each patient in the CHiP Study, the level of calorific intake (the treatment) was measured daily from study entry (randomization) until the relevant clinical outcome was recorded (discharge from the PICU or death while in the PICU). We restricted the follow-up data used in this analysis to the first 7 days postrandomization, since the majority of patients were discharged from the PICU by this time point.

In our analysis, we denote time by t=0, ...,T+1, where T+1=7 is the end of follow-up. At each time point, the patient’s feeding status is represented by the binary variable $A_{t}$, while confounders are denoted by the multidimensional variable $Z_{t}$. The first measurement of the time-varying confounders and the vector of baseline confounders are jointly denoted by $Z_{0}$. Mechanical ventilation, renal replacement therapy, infection, and randomization arm are binary variables, while the RACHS-1 and vasoactive inotrope scores, weight, height, and age are continuous.

$M_{t}$ and $Y_{t}$ indicate whether, by the end of time period *t*, a patient has died $\left(M_{t}= 1\right)$ or has been discharged alive from the PICU $\left(Y_{t}= 1\right)$. We use overbars to denote histories; for example, treatment history is denoted by ${\overset{¯}{A}}_{t}=(A_{0}, ...,\;A_{t})$. We assume that the observed data are *n* independent and identically distributed copies of $O=(Z_{0},A_{0},Y_{1},M_{1},Z_{1},A_{1}, ...,\;A_{T},Y_{T+1})∼P_{o}$, where $P_{o}$ is the true underlying distribution from which the data are drawn and where, for notational convenience, we assume that the values of variables after death or discharge are deterministically equal to their last observed values. The ordering of the elements of *O* represents their assumed causal ordering. (See Web Appendix 1, available at https://academic.oup.com/aje, for the causal model.) For example, the baseline covariates $\left(Z_{0}\right)$ precede the first instance of feeding $\left(A_{0}\right)$, which precedes whether the patient is discharged by the end of the first day $\left(Y_{1}\right)$.

We denote the history of the confounders, discharge status, and death with a single vector ${\overset{¯}{L}}_{t}=({\overset{¯}{Z}}_{t},{\overset{¯}{Y}}_{t},{\overset{¯}{M}}_{t})$ that will be referred to as “covariates.” Death is treated as a competing event for PICU discharge: If $M_{t}= 1$, then any subsequent $Y_{t\prime }=0$ for t′>t.

### Formulating the interventions of interest

We consider 2 types of longitudinal interventions: static treatment regimes and dynamic treatment regimes. Let the vector $\bar{a}=(a_{0},a_{1}, ...,\;a_{T})$ denote a longitudinal feeding regime, defined up to the last period before the end of follow-up. The elements of this vector, $a_{t}$, define the feeding intervention, with static and dynamic regimes differing in how $a_{t}$ is specified. For a static regime, $a_{t}$ is a prespecified constant for each *t*. For example, the static regime “never feed” sets $a_{t}$ to 0 in each *t*, resulting in the treatment regime a= (0, 0, ..., 0), while the static regime “feed from day 3” would be defined as a= (0, 0, 1, ..., 1).

For dynamic regimes, $a_{t}$ is set by a decision rule. We define $d_{t}$ as a function that incorporates information available on a subject up to time *t*, such as some subset of the covariate history, denoted ${\bar{V}}_{t}$. We use $d\left({\overset{¯}{V}}_{t}\right)$ to denote the vector of interventions required by regime *d* from time 0 to time *t*, given the realized covariate history. Here, we specify a dynamic treatment regime in which clinicians are required to feed a patient on each day that he or she is not mechanically ventilated $(Z_{t,1}= 1)$.

Clinical guidelines may not require the intervention to start on the first day and could allow delaying the start of the intervention. For example, the regime “feed by the third day” leaves the treatment values to be random for 2 days and then requires feeding from day 3 onward. This regime is denoted by ${\bar{a}}_{2:T}=(A_{0},A_{1}, 1, ..., 1)$, where $A_{0}$ and $A_{1}$ are the observed levels of feeding on the first 2 days.

Throughout, we consider regimes that implicitly only assign a feeding intervention up to the time of discharge or death. Thus, a subject who followed a regime of interest up to death or discharge, according to our definition, would continue to follow this regime up to time T+1. To simplify notation, in the sections that follow, we refer to the counterfactual interventions generally as $d=(d_{0}, ...,\;d_{T})\in D$, where D is the set of regimes of interest, and note that this notation includes static regimes as special cases of dynamic regimes.

### Target causal parameter and identifying assumptions

The counterfactual discharge status at time *t* that would have been observed under a given feeding regime *d* is denoted by $Y_{t}^{d}$. Our causal parameter of interest is the intervention-specific mean outcome, the expected discharge status by a selected time $t^{⁎}$, under a given regime *d*, where $t^{⁎}\;= 1, ...,\;T+1$:

$$\psi_{d,t^{⁎}}=E[Y_{t^{⁎}}^{d}].$$

$\psi_{d,t^{⁎}}$ can be interpreted as the counterfactual cumulative risk of discharge by day $t^{⁎}$ if all subjects had followed a given regime.

In order to identify $\psi_{d,t^{⁎}}$ from the observed data, the following assumptions are required (2):

The *sequential randomization assumption* states that, conditional on the observed treatment and confounder history, the potential outcome is independent of treatment status in each preceding time period,

$$Y_{t^{⁎}}^{d}⊥A_{t}|{\overset{¯}{L}}_{t},{\overset{¯}{A}}_{t-1}=d\left({\bar{V}}_{t-1}\right),$$

for $t\;= 0, ...,\;t^{⁎}-1$, and d∈D. This assumption requires that a sufficiently rich set of confounders are measured, so that it can be assumed that conditional on observed covariates, and following the regime of interest, the feeding decision at time *t* is “at random.”

The *positivity assumption* requires that for each feeding regime *d*, in each period *t* before the final time period of interest $t^{⁎}$, patients must have a positive probability of following that regime, conditional on having followed it up to that time point, for any combination of observed covariate history:

$$Pr[A_{t}=d\left({\overset{¯}{V}}_{t}\right)|\;{\overset{¯}{L}}_{t},{\overset{¯}{A}}_{t-1}=d({\overset{¯}{V}}_{t-1})] > 0,$$

for $t\;= 0, ...,\;t^{⁎}-1$.

### Estimation: IPTW

In this section we describe the IPTW estimator, the g-computation estimator, and the longitudinal TMLE estimator. We focus on implementation of the estimators for interventions that start on the first day. For interventions with a delayed start, we provide small modifications of the estimators in Web Appendix 2.

IPTW estimates the intervention-specific mean outcome of a treatment regime by reweighting the observed outcomes in the subset of the study sample who followed the regime (11). We denote the probability of a subject’s following a regime of interest $d_{t}$ at time *t*, given his/her covariate and treatment history, with

$$g_{t}=Pr[A_{t}=d_{t}\left(\overset{¯}{V_{t}}\right)|\;{\overset{¯}{A}}_{t-1}=d\left({\overset{¯}{V}}_{t-1}\right),{\bar{L}}_{t}],$$

and the cumulative conditional probability of following regime *d* through time $t^{⁎}-1$ as

$$g_{0:t^{⁎}-1}=\prod_{t=0}^{t^{⁎}-1}g_{t}.$$

The stabilized Horvitz-Thompson IPTW estimator (14, 40) of the cumulative risk of discharge by period $t^{⁎}$ under treatment regime *d* is based on estimation of the following quantity:

$$\begin{array}{c} \frac{E[Y_{t^{⁎}}\times I({\bar{A}}_{t^{⁎}-1}=d\left({\bar{V}}_{t^{⁎}-1}\right))/g_{0:t^{⁎}-1}]}{E[I({\bar{A}}_{t^{⁎}-1}=d\left({\bar{V}}_{t^{⁎}-1}\right))/g_{0:t^{⁎}-1}]}, \end{array}$$

where $I({\overset{¯}{A}}_{t^{⁎}-1}=d\left({\overset{¯}{V}}_{t^{⁎}-1}\right))$ indicates whether a patient has followed the treatment regime *d* up to one period before the final period of interest. Implementation is based on estimating $g_{t}$ for $t\;= 0, ...,\;t^{⁎}-1$, plugging in these estimates, and taking the empirical mean of the numerator and denominator.

Drawbacks of the IPTW estimator include reliance on consistent estimation of the treatment mechanism, as well as susceptibility to violations and near violations of the positivity assumption, resulting in unstable estimates (see, for example, Petersen et al. (15)). In the next section, we describe the longitudinal TMLE estimator, a doubly robust estimator which can improve on the properties of the IPTW estimator by using information not only on the treatment mechanism but also on the outcome-confounder(s) relationship.

### Estimation: longitudinal TMLE

#### The conditional expectation representation of the g-computation formula

Longitudinal TMLE (21) uses the identifiability result established by the g-computation formula (2). In short, the g-computation formula expresses the intervention-specific mean outcome as a function of the conditional distributions of the outcome and the time-varying confounders, given the past, among subjects who followed the regime of interest. For discrete-valued confounders, this can be written as follows:

$$\begin{array}{c} E\left[Y_{t^{⁎}}^{d}\right]=\sum_{{\overset{¯}{l}}_{t^{⁎}-1}}E[Y_{t^{⁎}}|{\overset{¯}{A}}_{t^{⁎}-1}=\;d\left({\overset{¯}{v}}_{t^{⁎}-1}\right),{\overset{¯}{L}}_{t^{⁎}-1}={\overset{¯}{l}}_{t^{⁎}-1}]\\ \;\times \prod_{t=0}^{t^{⁎}-1}Pr(L_{t}=l_{t}|{\overset{¯}{A}}_{t-1}=d\left({\overset{¯}{v}}_{t-1}\right),{\overset{¯}{L}}_{t-1}={\overset{¯}{l}}_{t-1}), \end{array}$$

where the summation is taken over all possible values ${\overset{¯}{l}}_{t^{⁎}-1}$ of the confounder history. Intuitively, the g-computation formula estimates the conditional expectation of the outcome under the treatment regime of interest and averages these expectations over the intervened-on distribution of the confounders—that is, the distribution that the confounders would take under the treatment regime of interest. Parametric g-computation (2, 41, 42) estimates the components of this formula directly, and it makes strong parametric assumptions due to the need to specify conditional densities or probabilities for each of the time-varying confounders (12, 43).

The g-computation formula can be rewritten as a series of iterated conditional expectations of the observed outcome (26, 44, 45):

(1) $$\begin{array}{c} E\left[{Y_{t^{⁎}}}^{d}\right]\;=E[\ldots E[E[E[Y_{t^{⁎}}|{\overset{¯}{A}}_{t^{⁎}-1}=\;d({\overset{¯}{V}}_{t^{⁎}-1}),{\overset{¯}{L}}_{t^{⁎}-1}]\;|\;\\ \;{\overset{¯}{A}}_{t^{⁎}-2}=d({\overset{¯}{V}}_{t^{⁎}-2}),{\overset{¯}{L}}_{t^{⁎}-2}]\;|\;\\ \;{\overset{¯}{A}}_{t^{⁎}-3}=d({\overset{¯}{V}}_{t^{⁎}-3}),{\overset{¯}{L}}_{t^{⁎}-3}]\ldots ], \end{array}$$

where the innermost expectation is the conditional distribution of the outcome, given the full treatment and confounder history, evaluated at the treatment values that would have been assigned according to the intervention of interest *d*. The second innermost expectation marginalizes over the intervened-on history of $L_{t^{⁎}-1}$, the next one over $L_{t^{⁎}-2}$, and so on, until the last expectation is taken over the empirical distribution of baseline confounders $L_{0}$, where $M_{0}= 0$ and $Y_{0}= 0$. We first briefly review how to obtain the target parameter using these iterative regressions and then describe how the longitudinal TMLE extends this approach.

#### Steps of g-computation using sequential regressions

##### Step 1: Regress the outcome on full treatment and confounder history.

First the innermost expectation of equation 1 is estimated:

$$E[Y_{t^{⁎}}|\;{\overset{¯}{L}}_{t^{⁎}-1},{\overset{¯}{A}}_{t^{⁎}-1}=d\left({\overset{¯}{V}}_{t^{⁎}-1}\right)].$$

We will refer to this quantity as ${\overset{¯}{Q}}_{t^{⁎}}$. This expectation can be estimated by regressing the outcome on past covariates and treatment variables—for example, using a logistic regression—and taking predictions at the treatment values corresponding to the intervention of interest.

##### Step 2: Take the previous predictions as the new outcome and regress on history up to $t^{⁎}-2$

The predictions from the previous step, ${\overset{¯}{Q}}_{t^{⁎}}$ are now taken as the new outcome and are regressed on confounders and treatment variables up to time period $t^{⁎}-2$. As before, predictions are generated for treatment values required by the regime $\overset{¯}{d}$, up to time period $t^{⁎}-2$. This expectation, ${\overset{¯}{Q}}_{t^{⁎}-1}$, corresponds to the second innermost expectation in equation 1. ${\overset{¯}{Q}}_{t^{⁎}-1}$ is marginal over the intervened-on distribution of the time-varying confounder $L_{t^{⁎}-1}$ but conditional on the time-varying confounders up to time period $L_{t^{⁎}-2}$.

##### Steps 3, 4, . . . to step $t^{⁎}$: Iterate step 2

Step 3 takes the predictions from step 2, ${\overset{¯}{Q}}_{t^{⁎}-1}$, regresses them on the treatment and confounder history up to $t^{⁎}-3$, and then takes predictions as described above, stored as ${\overset{¯}{Q}}_{t^{⁎}-2}$. This step is iterated until the last step, where the expectation is only conditional on the baseline covariates:

$${\overset{¯}{Q}}_{1}=E[{\overset{¯}{Q}}_{2}|{\overset{¯}{L}}_{0},{\overset{¯}{A}}_{0}=d\left(V_{0}\right)].$$

##### Step $t^{⁎}+1$: Average over the empirical distribution of the baseline covariates

By averaging ${\overset{¯}{Q}}_{1}$ over the empirical distribution of $L_{0}$, the g-computation estimator for the intervention-specific mean is obtained as ${\overset{¯}{Q}}_{0}=E\left[{\overset{¯}{Q}}_{1}\right]$.

Each ${\overset{¯}{Q}}_{t}$ can be obtained using a regression—for example, a linear or logistic regression. This approach offers substantial advantages over the parametric g-computation approach, by avoiding the need to estimate the conditional density of each time-varying confounder. However, estimating these iterative regressions well can be challenging, and the approach remains susceptible to bias due to misspecification. Bang and Robins (26) proposed a doubly robust and semiparametric efficient version of this sequential regression estimator based on including an additional, “clever” covariate that uses information from the treatment assignment mechanism. It has subsequently been suggested to move this clever covariate to a weight, an approach that improved performance in the face of practical positivity violations (46). The resulting estimator is doubly robust in the sense that if either the treatment mechanisms or the sequential regressions are estimated consistently, then the estimator is consistent. If both are estimated consistently, it is efficient in a semiparameteric model that makes assumptions, if any, only on the treatment mechanism (26). Van der Laan and Gruber (21) subseqently placed this estimator in the general TMLE framework. The general idea behind this TMLE is that it is a 2-step estimator: First the conditional expectation of the outcome is estimated, and then this estimate is updated using information from the treatment assignment mechanism, targeted in a way that it reduces bias for the parameter of interest. Longitudinal TMLE performs the update step at each stage of the sequential regressions, as we summarize below.

#### The update step of the TMLE estimator

${\overset{¯}{Q}}_{t^{⁎}}$ is defined and estimated as in step 1 of the g-computation approach. This initial estimate is then updated by perturbing the initial fit ${\overset{¯}{Q}}_{t^{⁎}}$ using a parametric submodel, defined as

$$\mathrm{logit}({\overset{¯}{Q}}_{t^{⁎}}^{1}\left(\varepsilon_{t^{⁎}}\right))=\mathrm{logit}\left({\overset{¯}{Q}}_{t^{⁎}}\right)+\varepsilon_{t^{⁎}}.$$

We estimate $\varepsilon_{t^{⁎}}$ by fitting a logistic regression of $Y_{t^{⁎}}$ on the intercept, using the prior predicted value of ${\overset{¯}{Q}}_{t^{⁎}}$ as an offset, and weights corresponding to $I({\bar{A}}_{t^{⁎}-1}=d\left({\bar{V}}_{t^{⁎}-1}\right))/g_{0:t^{⁎}-1}$, an indicator of whether a subject has followed the regime of interest up to the previous time period divided by the predicted probability of having done so. The estimated $\varepsilon_{t^{⁎}}$ is then used to update the initial estimate, which is stored as ${\overset{¯}{Q}}_{t^{⁎}}^{1}$, and will be used as the new outcome for the next iteration.

This update is performed after each step of the sequential regressions, described for the g-computation estimator. The regression and update steps are iterated until the last step, in which the updated expectation ${\overset{¯}{Q}}_{1}^{1}$ is only conditional on the baseline covariates. Analogous with the last step of the g-computation estimator, the TMLE estimator for the intervention-specific mean is obtained as ${\overset{¯}{Q}}_{0}^{1}=E\left[{\overset{¯}{Q}}_{1}^{1}\right]$.

The consistency of the estimator relies on consistent estimation of either the treatment mechanism or the iterated conditional regressions, while its efficiency relies on consistent estimation of both. In practice, often both components are expected to be misspecified when fixed, parametric models such as logistic regressions are used. Machine learning or data-adaptive approaches are thus advocated for estimation of both (19). We use the Super Learner (47), a machine learning algorithm that uses cross-validation to find the optimal weighted convex combination of multiple candidate prediction algorithms, for estimating both the treatment assignment mechanism and the sequential regressions (see Web Appendix 3 for more details).

### Implementation

We implement the IPTW, g-computation, and TMLE estimators described above to estimate the cumulative probability of PICU discharge by the end of days 1–7, under a range of prespecified static treatment regimes (“never feed,” “feed from day 1, 2, 3, . . .7”), static regimes over limited time periods (“feed by day 2, 3, . . .7”), and the dynamic regime “feed when off ventilation.”

We use the Super Learner to estimate the treatment assignment mechanism and the sequential regressions, and we use these models to construct the 3 estimators. Among the Super Learner candidates, we include an intercept model, a main-terms model, a logistic regression model with all possible 2-way interactions in the linear predictor, a stepwise logistic regression model, generalized additive models (48), a Bayesian generalized linear model with main terms in the linear predictor (49), a LASSO model (50), a boosting algorithm (51), and a neural networks algorithm (52). We specify 10-fold cross-validation (47). We fit separate models for the treatment assignment mechanism for each period, while assuming that treatment decisions are influenced only by treatment and confounder values in the 2 most recent periods. The regressions carried out to obtain the conditional probability of treatment and the iterative regressions and the update steps of the TMLE are only run among those children who remain alive and not discharged. We contrast these estimates with “naive” estimates, taken as the simple proportion of discharge status among those who follow a given regime.

The 95% confidence intervals are based on an estimate of the empirical influence function (53, 54) of the IPTW and TMLE estimators. For the g-computation estimator, no influence-function-based approach for inference is readily available, and the point estimates are reported without 95% confidence intervals. While the nonparametric bootstrap represents an alternative approach to variance estimation, when Super Learner is used to conduct the sequential regressions without subsequent targeting, bootstrapping can impose a substantial computational burden while still failing to provide valid inference. The availability of an influence-curve-based variance estimator compatible with machine learning approaches is thus an additional attractive feature of TMLE. The methods are implemented using the package “ltmle” in R, version 0.9-9 (R Foundation for Statistical Computing, Vienna, Austria) (55, 56), which incorporates the Super Learner R package (57). We provide the main R functions used for the analysis in Web Appendix 4.

## RESULTS

**Figure 1.**
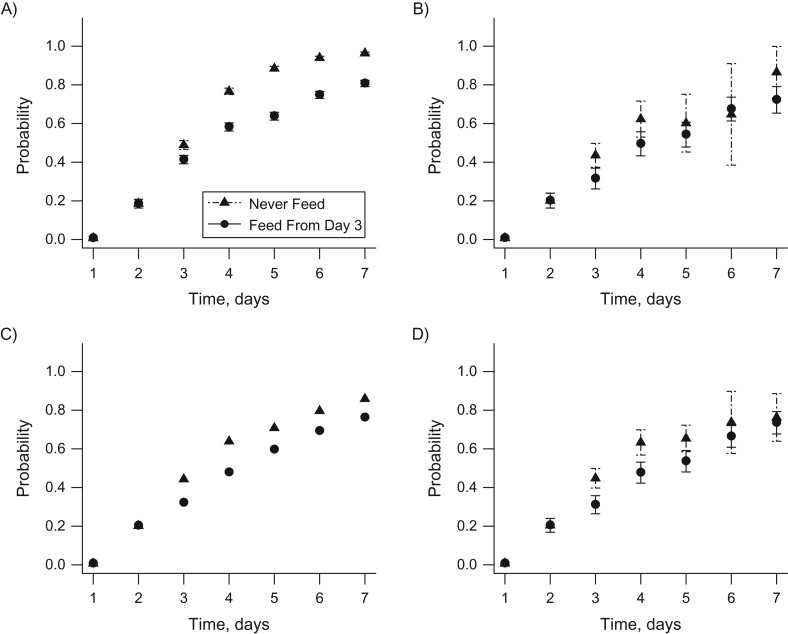
Estimated cumulative probabilities of discharge from the pediatric intensive care unit by the end of days 1–7 for the feeding regime “never feed” versus “feed from day 3,” based on data from the Control of Hyperglycemia in Paediatric Intensive Care (CHiP) Study (32), 2008–2011. A) Unadjusted estimates and 95% confidence intervals (bars); B) inverse probabaility of treatment weighting estimates and 95% confidence intervals (bars); C) G-computation estimates; D) targeted maximum likelihood estimates and 95% confidence intervals (bars). The *x* axis shows time in days, while the *y* axis displays the estimated counterfactual probability of discharge from the pediatric intensive care unit, by the end of a given day, for a given regime. The 95% confidence intervals for the g-computation estimates are not reported.

A total of 706 children were included in the study sample. Table 1 shows the number of patients who were still in the PICU at each time point and, among those, the number of patients receiving less than 20% of their daily caloric target (not fed) and those receiving at least 20% (fed). Table 2 shows the numbers of patients observed to have followed each static regime of interest, up to a given day. Figure 1 contrasts the static regimes “never feed” and “feed from day 3,” showing the naive estimates, not adjusted for any observed confounders (Figure 1A), and the IPTW (Figure 1B), g-computation (Figure 1C), and TMLE estimates (Figure 1D). See Web Table 1 for an illustrative calculation of the naive estimates for patients who followed the regime “feed from day 3.”

**Table 1.** Patient Flow on Each Hospital Day, by Treatment Status and Outcome (*n* = 706), CHiP Study, 2008–2011

Hospital Day	In PICU	In PICU, Fed	In PICU, Not Fed	Cumulative No. of Patients Deceased	Cumulative No. of Patients Discharged
1	706	28	678	0	0
2	701	260	441	0	5
3	597	387	210	0	109
4	434	340	94	0	272
5	325	278	47	3	378
6	248	222	26	5	453
7	188	169	19	7	511

Abbreviations: CHiP, Control of Hyperglycemia in Paediatric Intensive Care; PICU, pediatric intensive care unit.

**Table 2.** Cumulative Numbers of Patients Whose Data Were Consistent With Each Static Feeding Regime (*n* = 706), CHiP Study, 2008–2011

Hospital Day	Feed From . . .							Never Feed
	Day 1	Day 2	Day 3	Day 4	Day 5	Day 6	Day 7	
1	28	678	678	678	678	678	678	678
2	24	241	442	442	442	442	442	442
3	24	220	254	270	270	270	270	270
4	22	212	237	205	197	197	197	197
5	21	205	232	195	178	173	173	173
6	21	202	226	192	176	165	165	165
7	21	200	223	186	175	164	163	162

Abbreviation: CHiP, Control of Hyperglycemia in Paediatric Intensive Care.

The naive estimates indicate a significantly higher probability of being discharged by each day for the “never feed” regime as compared with the “feed from day 3” regime. For example, the probability of discharge by the end of day 5 is 0.88 (95% confidence interval (CI): 0.87, 0.90) for “never feed,” in contrast to the significantly lower estimate of 0.64 (95% CI: 0.62, 0.66) for “feed from day 3.” Adjustment for baseline and time-varying confounders shifts the estimated probability of discharge in each time period downwards and reduces the difference between the two regimes. Using TMLE, we estimated a 0.66 probability of discharge by the end of day 5 for children who were never fed (95% CI: 0.59, 0.72) and a 0.53 probability for those who were fed from day 3 (95% CI: 0.48, 0.59). The TMLE, IPTW, and g-computation estimators produced similar point estimates, while TMLE generated narrower 95% confidence intervals than the IPTW estimator. For example, the probability of discharge by the end of day 5 for the “feed from day 3” regime was estimated to be 0.54 (95% CI: 0.47, 0.60) using IPTW and 0.59 using g-computation. The smallest estimated cumulative probability of following a given regime, across all of the regimes considered, was more than 0.05, so no weight truncation was used.

**Figure 2.**
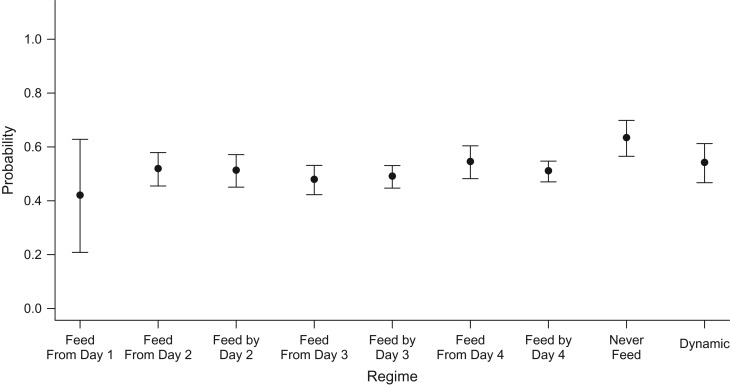
Estimated cumulative probabilities of discharge from the pediatric intensive care unit by the end of day 4, based on data from the Control of Hyperglycemia in Paediatric Intensive Care (CHiP) Study (32), 2008–2011. The *x* axis displays the regimes compared, while the *y* axis displays the targeted maximum likelihood estimates and corresponding 95% confidence intervals (bars) for the counterfactual probabilities of discharge by the end of day 4 for a given feeding regime. “Dynamic” = feed when off mechanical ventilation.

Focusing on the cumulative probability of discharge by day 4, Figure 2 contrasts the intervention-specific mean estimates across all regimes distinguishable by this time point, estimated by TMLE. The estimated probability for the static regime “feed from day 1” is 0.42, with the widest 95% confidence interval among all regimes (95% CI: 0.21, 0.62), which can be explained by the low number of patients following this regime. Regimes requiring starting feeding from the third day onward or by the third day, compared with starting on the second day, have lower expected probabilities of discharge; however, the 95% confidence intervals overlap. As before, the regime “never feed” has the most favorable expected outcomes (TMLE producing an estimated probability of discharge of 0.63 (95% CI: 0.57, 0.79)); however, the probability of discharge under this regime is not statistically significantly different from that under the other regimes.

## DISCUSSION

We implemented a doubly robust approach, TMLE, to contrast the counterfactual probability of being discharged alive from the PICU under a set of static and dynamic longitudinal feeding regimes in a population of critically ill children. While the unadjusted estimates showed a significant difference in discharge probabilities between the treatment regimes “start feeding from the third day” and “never feed,” after adjustment most of this difference disappeared. TMLE estimators produced narrower confidence intervals than IPTW, as predicted by theory (17), while influence-curve-based confidence intervals for g-computation estimators were not readily available. We found no strong evidence that high levels of caloric intake may lead to adverse health outcomes in critically ill children. While in this paper the 3 statistical approaches led to similar conclusions, depending on the setting, TMLE may give substantially different results from estimation methods that are not doubly robust or do not exploit data-adaptive model selection (see, for example, Decker et al. (27) for an application and Schnitzer et al. (28) for simulation evidence).

An observational analysis of data from a clinical trial enabled us to investigate the impact of alternative longitudinal feeding practices on clinical outcomes. We contributed to the literature on application of longitudinal causal methods in the PICU setting, where, due to the fast-changing prognosis of patients and subsequently updated treatment decisions, time-dependent confounding is an important concern (58). Using clinical judgement on meaningful longitudinal treatment regimes, we selected a range of static and dynamic interventions which were supported by the data, and asked new causal questions. While data collected in a clinical trial can provide advantages, such as regular intervals of follow-up, measurement of a rich set of observed and time-varying confounders, and little missing data, the approach taken generalizes to settings of observational data.

This paper further provides a demonstration of the application of TMLE for longitudinal static and dynamic regimes and highlights how it builds on alternative approaches such as IPTW and g-computation, under the challenging circumstances of a real-world comparative effectiveness study: a large number of covariates to adjust for and a medium-sized sample. Application of the methods to address an unanswered clinical question of high relevance in intensive care raised several methodological issues. Beyond static and dynamic treatment regimes, we also considered interventions with a delayed start (e.g., “feed by day 3”) motivated by clinical practice. The availability of daily measurements of time-varying confounders resulted in high dimensionality of observed covariates to adjust for. Informed by clinical judgement, we assumed that the decision as to whether to feed on a given day is influenced only by observed characteristics measured on the given day and on the previous day. To deal with the challenge of model specification, we used the data-adaptive algorithm Super Learner.

Each of the methods applied here relies on the assumption that in each period, all time-constant and time-varying confounders that can influence treatment assignment and the outcome are observed. While the CHiP trial recorded data on a rich set of covariates, a patient’s prognosis changes quickly over time, and the observed time-varying characteristics (mechanical ventilation, renal replacement, inotrope score) may not capture all confounders. In particular, if a clinician expects a patient to be discharged from the PICU soon, he or she may temporarily decrease or not initiate enteral feeding, to prevent delay in discharge. Further research using methods to analyze the sensitivity of the parameter estimates to the presence of unobserved confounders is therefore warranted (59).

In summary, this paper illustrates that existing data sources, such as well-conducted randomized controlled trials, can be exploited to address important questions of clinical decision-making beyond those originally posed. A wider use of appropriate causal methods could add to the understanding of the advantage of alternative sequencing of time-varying treatments and could provide estimates of the effectiveness and cost-effectiveness of realistic treatment strategies.

## Supplementary Material

Web Material

## Abbreviations

CHiP: Control of Hyperglycemia in Paediatric Intensive Care
CI: confidence interval
IPTW: inverse probability of treatment weighting
PICU: pediatric intensive care unit
RACHS-1: Risk Adjustment for Congenital Heart Surgery
TMLE: targeted maximum likelihood estimation
