# Supplementary material for: Estimating the Comparative Effectiveness of Feeding Interventions in the Pediatric Intensive Care Unit: A Demonstration of Longitudinal Targeted Maximum Likelihood Estimation
Source: Am J Epidemiol. 2017 Jun 24;186(12):1370–9. doi: 10.1093/aje/kwx213 (PMC5860499; doi:10.1093/aje/kwx213)
Supplement: Web Material [file kwx213kreifwebmaterialfinal.pdf]

Web material for the article "Estimating the comparative effectiveness of feeding interventions in the paediatric intensive care unit: a demonstration of longitudinal targeted maximum likelihood estimation"

Noémi Kreif *[1], Linh Tran[2], Richard Grieve[3,4], Bianca deStavola[3,5], Robert C Tasker[6,7], and Maya Petersen[2]

[1]Centre for Health Economics, University of York, York, UK

[2]Division of Biostatistics, UC Berkeley School of Public Health, Berkeley, CA, USA

[3]Centre for Statistical Methodology, London School of Hygiene and Tropical Medicine, London, UK

[5]Department of Medical Statistics, London School of Hygiene and Tropical Medicine, London, UK

[4]Department of Health Services Research and Policy, London School of Hygiene and Tropical Medicine, London, UK

[6]Department of Anesthesiology, Perioperative and Pain Medicine, Division of Critical Care, Boston Children's Hospital, Boston, MA, USA

[7]Department of Neurology, Boston Children's Hospital, Boston, MA, USA

October 23, 2017

Web Appendix 1

Causal model

We assume the following nonparametric structural equation model, $\mathcal{M}$:

$Y_t = f_{Y_t}(\overline{Y}_{t-1}, \overline{M}_{t-1}, \overline{Z}_{t-1}, \overline{A}_{t-1}, U_{Y_t})$, for $t = 1, ..., T+1$

$M_t = f_{M_t}(\overline{Y}_t, \overline{M}_{t-1}, \overline{Z}_{t-1}, \overline{A}_{t-1}, U_{M_t})$, for $t = 1, ..., T$

$Z_t = f_{Z_t}(\overline{Y}_t, \overline{M}_t, \overline{Z}_{t-1}, \overline{A}_{t-1}, U_{Z_t})$, for $t = 0, ..., T$

$A_t = f_{A_t}(\overline{Y}_t, \overline{M}_t, \overline{Z}_t, \overline{A}_{t-1}, U_{A_t})$, for $t = 0, ..., T$,

where $U_t = (U_{Y_t}$, U_{M_t}, U_{Z_t} and $U_{A_t})$, $t = 0, ..., T+1$ are unmeasured exogenous random variables from some underlying probability distribution P_U. This causal model specifies how each of the variables in the data are generated, with randomness arising only from from the exogenous variables U. For example, the outcome at a given time period, Y_t is a deterministic function of the full history of treatment and confounder values, and a random error. Y_t and M_t are also functions of previous values of Y and M, encoding the information that after a patient is discharged, she always remains discharged, but if a patient dies in a given time period, she remains dead, and can never be discharged. More generally, after an event of death or discharge, all the processes become degenerate, and for notational convenience we assume that they take the last value observed. For notational convenience the causal model allows for Y_0, and M_0, which are both assumed to take value 0 (at baseline no one is dead or discharged), and Z_{-1}, A_{-1} which are assumed to be empty vectors.

*corresponding author details: Noemi Kreif, Centre for Health Economics, University of York, Heslington, York, YO10 5DD, UK. Tel: work +44 (0)1904 321401 e-mail: noemi.kreif@york.ac.uk.

Web Appendix 2

Modification of the estimators for the static regimes with delayed start

For regimes where the intervention starts with a delay, such as 'feed by day k', corresponding to an intervention beginning at $t = k - 1$, the A_t nodes denoting feeding prior to time $k - 1$ are treated as non-intervention nodes or 'covariates'. As a result, the baseline covariates (measured prior to the first intervention node A_{k-1}) consist of

$$(Z_0, A_0, ..., Y_{k-1}, M_{k-1}, Z_{k-1}).$$

When estimating $E[Y^d_{t^*}], t^* = k, ..., T+1$, there are thus a total of $t^* - (k-1)$ rather than t^* intervention nodes, and a corresponding number of components to the regimes of interest $(d(\bar{V}_t) = d_{k-1}(\bar{V}_{k-1}), ..., d_{t^*-1}(\bar{V}_{t^*-1}))$.

The IPTW, g-computation, and TMLE estimators are modified accordingly. First, the indicator of following a regime of interest through time $t^* - 1$, $I(\bar{A}_{t^*-1} = d(\bar{V}_{t^*-1}))$, used in the numerator of the weights for the IPTW and TMLE estimators, corresponds to an indicator of following the regime from time $k - 1$ to $t^* - 1$. (In other words, all subjects follow the regime of interest before $k - 1$). Second, the cumulative probability of following the regime of interest, used in the denominator of the weights for the IPTW and TMLE estimators, is now based on a product of time point-specific probabilities of continuing to follow the regime beginning at time $k - 1$:

$$g_{k-1:t^*-1} = \prod_{t=k-1}^{t^*-1} g_t(A_t = d_t(\bar{V}_t) | \bar{A}_{t-1} = d(\bar{V}_{t-1}), \bar{L}_t).$$

Finally, the presence of fewer intervention nodes implies that the longitudinal g-formula can be expressed using $t^* - (k-1)$ rather than t^* iterated conditional expectations; one conditional expectation is needed for each intervention node.

Web Appendix 3

Super Learning estimation of the treatment and outcome mechanism

The Super Learner (1) is a machine learning algorithm that uses cross validation to find the optimal weighted convex combination of multiple candidate prediction algorithms. The algorithms are pre-selected by the analyst, potentially including parametric and non-parametric regression models, as well as a range of machine learning approaches. Asymptotically, the Super Learner algorithm performs as well as the best possible combination of the candidate estimators, assuming that none of the candidates in the library is a correctly specified parametric model; in the latter case it achieves almost parametric rate of converge (see (2) and (3) for details). Beyond its use for prediction (4; 5), it has been used for estimating the propensity score and the outcome model to obtain causal parameters (for example, (6; 7; 8)), and has been shown to reduce bias from model misspecification (9; 10; 11).

Web Appendix 4

Main R functions used in the analysis

```
#####  deterministic Q function ############################

# objective: set Q to 1 deterministically if any prior L2=1

my.det.Q.fun <- function(data, current.node, nodes, called.from.estimate.g) {
  dnodes <- grep("L2", names(data))
  if (! any(dnodes < current.node)) { # outputs FALSE if there is no death node
      before currnt Anode
    return(NULL)
  }
  dnodes <- dnodes[dnodes < current.node] # only look at death nodes before current
       node
  dnodes.is1 <- data[, dnodes, drop=FALSE] == 1 & !is.na(data[, dnodes, drop=FALSE
      ]) # true if dnode is 1 (and not NA)
  dead <- apply(dnodes.is1, 1, any)
  return(list(is.deterministic=dead, Q.value=0))
}
#### MAIN FUNCTION ACTUALLY CALLING TMLE, it takes different arguments for
    different kinds of interventions
#### It calculates iptw, tmlw and gcomp, for 2 interventions. One is never feed (
    static), this stays fixed, I call it control.
```

```
#### The other one is a "treatment" regime, either static, or with delayed
    intervention ( v ) needs to be specifed, or dynamic.

#### Dynamic intervention is currently a rule based on the presence of mechanical
    ventilation each day


my.ltmle.contrast <- function(time, treatment, adjusted, sl) {
  d.time <- pick_data(time)
  n <- nrow(d.time$d)

  abar.static.0 <- rep(0, time)
  if (identical(treatment, "dynamic")) {
    ### dynamic 1: mech vent ######
    abar.1 <- as.matrix(d.time$d[, paste0("L4.", 0:(time - 1))])
    intervene.time <- 1:time
  } else {
    set.to.1 <- if (treatment$day <= time) treatment$day:time else NULL
    if (treatment$delay) {
      intervene.time <- set.to.1
    } else {
      intervene.time <- 1:time
    }

    ### static or delayed: intervention starts on day
    abar.1 <- matrix(0, nrow = n, ncol = time)
    abar.1[, set.to.1] <- 1
  }
  abar.1.subset <- abar.1[, intervene.time, drop = FALSE]
```

```
if (adjusted) {
  my.qform <- qform.generate(time)
  my.gform.0 <- gform.generate(time)
  my.gform.1 <- my.gform.0[intervene.time]
} else {
  my.qform <- qform.generate.unadjusted(time) # intercept only for Q
  my.gform.0 <- matrix(0, nrow=n, ncol=time) #set P(A=0)=1; unadjusted estimates
      are reported by setting the g matrix to 1s
  my.gform.1 <- abar.1.subset
}

result.list <- list()
for (gcomp in c(FALSE, TRUE)) {
  # run ltmle for control (always static)
  result.0 <- ltmle(d.time$d, Anodes=d.time$my.A.nodes, Lnodes=d.time$my.L.nodes,
       Ynodes=d.time$my.Y.nodes, abar=abar.static.0, SL.library=sl, estimate.time=
      FALSE, survivalOutcome=TRUE, variance.method='ic',deterministic.Q.function=
      my.det.Q.fun,gform=my.gform.0,Qform=my.qform, gcomp=gcomp)

  # run ltmle for treated
  result.1 <- ltmle(d.time$d, Anodes=d.time$my.A.nodes[intervene.time], Lnodes=d.
      time$my.L.nodes, Ynodes=d.time$my.Y.nodes, abar=abar.1.subset, SL.library=sl
      , estimate.time=FALSE, survivalOutcome=TRUE, variance.method='ic',
      deterministic.Q.function=my.det.Q.fun,gform=my.gform.1, Qform=my.qform[
      intervene.time], gcomp=gcomp)

  result.list <- c(result.list, GetAllResults(result.0, result.1, gcomp))
}
```

```
  return(result.list)
}

GetConfInt <- function(result, estimator) {
  c(summary(result, estimator)$treatment$estimate, summary(result, estimator)
      $treatment$CI)
}

GetResults <- function(result.0, result.1, estimator) {
  x <- list(GetConfInt(result.0, estimator), GetConfInt(result.1, estimator))
  names(x) <- paste("est", c("ctrol", "tr"), estimator, sep = ".")
  return(x)
}

GetAllResults <- function(result.0, result.1, gcomp) {
  if (gcomp) {
    GetResults(result.0, result.1, "gcomp")
  } else {
    c(GetResults(result.0, result.1, "tmle"), GetResults(result.0, result.1, "iptw
        "))
  }
}
```

Web Table 1: Patient flow and unadjusted estimates: regime 'feed from day 3'

Hosp day	In PICU (t)	In PICU & follows (t))	Dischg event (t+1)	Death (t+1)	Stops following (t+1)	Cum. follows (t)	Cum. discharge (t)	Prob dischg by t+1 |follows t
1	706	678	5	0	236	678	5	0.007
2	701	437	77	0	265	442	82	0.186
3	597	172	23	0	17	254	105	0.413
4	434	132	33	0	5	237	138	0.582
5	325	94	10	0	6	232	148	0.638
6	248	78	21	0	3	226	169	0.748
7	188	54	11	0	0	223	180	0.807

Cumulative discharge is calculated amongst those whole followed the rule. The last column corresponds the unadjusted estimates reported in Figure 1.

References

[1] van der Laan MJ, Dudoit S. Unified cross-validation methodology for selection among estimators and a general cross-validated adaptive epsilon-net estimator: Finite sample oracle inequalities and examples; 2003. Working Paper 130. Available from: `http://biostats.bepress.com/ucbbiostat/paper130`.

[2] Van der Laan MJ, Polley EC, Hubbard AE. Super learner. Statistical applications in genetics and molecular biology. 2007;6(1):1–21.

[3] Van der Laan MJ, Rose S. Targeted learning: causal inference for observational and experimental data. Springer Science & Business Media; 2011.

[4] Polley EC, van der Laan MJ. Super learner in prediction; 2010. Working Paper 266. Available from: `http://biostats.bepress.com/ucbbiostat/paper266`.

[5] Rose S. Mortality risk score prediction in an elderly population using machine learning. American journal of epidemiology. 2013;177(5):443–452.

[6] Eliseeva E, Hubbard AE, Tager IB. An Application Of Machine Learning Methods To The Derivation Of Exposure-Response Curves For Respiratory Outcomes;. Working Paper 309. Available from: `http://biostats.bepress.com/ucbbiostat/paper309`.

[7] van der Laan MJ, Luedtke AR. Targeted learning of an optimal dynamic treatment, and statistical inference for its mean outcome; 2014. Working Paper 329. Available from: `http://biostats.bepress.com/ucbbiostat/paper329`.

[8] Gruber S, Logan RW, Jarrín I, Monge S, Hernán MA. Ensemble learning of inverse probability weights for marginal structural modeling in large observational datasets. Statistics in medicine. 2015;34(1):106–117.

[9] Porter KE, Gruber S, van der Laan MJ, Sekhon JS. The relative performance of targeted

maximum likelihood estimators. The international journal of biostatistics. 2011;7(1):1–34.

[10] Kreif N, Gruber S, Radice R, Grieve R, Sekhon JS. Evaluating treatment effectiveness under model misspecification: a comparison of targeted maximum likelihood estimation with bias-corrected matching. Statistical methods in medical research. 2016;25(5):2315–2336.

[11] Pirracchio R, Petersen ML, van der Laan M. Improving Propensity Score Estimators' Robustness to Model Misspecification Using Super Learner. American journal of epidemiology. 2015;181(2):108–119.
